# Ice

**Published:** 1879-05

**Authors:** 


To a person burning up with internal fevers ice is a comfort beyond expression.

Swallowing ice freely in small lumps is the chief treatment in inflammation of the stomach.

The constant application of ice, pounded fine, and enveloping the head with it by means of a cushion, or other contrivance, is the most reliable remedy for that dangerous malady, inflammation of the brain, which so often sends its victim to the grave in a few days, or to that living death, the mad-house!!

In all inflammations, whether internal or external, ice diminishes rapidly the size of the blood-vessels, and thus relieves the pain they give when thus swollen by their pressing against the nerves which are always in the neighborhood of the arteries of the system.

Diphtheria, and some of the worst of other forms of sore throat, has been arrested in a very short time by pounding a piece of ice in a bag, then laying the head back, take the lumps and swallow them continuously until relieved, allowing them to be detained in the throat as long as possible, there to melt.

In all forms of diarrhea and dysentery, where there is great thirst, the gratification of which by drinking any liquid increases the malady, are promptly controlled, and in many cases perfectly cured, by simply swallowing as large lumps of ice as possible.

Epilepsy itself, one of the most uncontrollable of human maladies, is said to be treated successfully in London by the application of ice to the spinal portion of the system.

A piece of ice laid on the wrist will often arrest profuse and dangerous bleeding of the nose.

In croup, water as cold as ice can make it, if applied freely and persistently to the throat, neck, and upper part of the chest with a sponge or cloth, often affords an almost miraculous relief, especially if followed by drinking copiously of ice-water, wiping the wetted parts perfectly dry, then wrapping the child closely up in dry flannels, allowing it to fall into a delightful and life-giving slumber.

These statements may induce the farmer to be at pains, if he does conclude to build an ice-house, to have it done in the most thorough manner and after the most approved pattern.

